# 

**Published:** 1894-03

**Authors:** 


					﻿Vol XIV.
MARCH, 1894.
NO. 3.
JPHE
pOMlEOPATHlC pHY^lClAJl,
A Monthly Journal of Homoeopathic Materia Medica
and Clinical Medicine.
"He it a freeman whom truth makes free, and all are slaves beside."
“ Seek the Truth: come whence it may, cost what it will."
EDITED AND PUBLISHED BY
WALTER M. JAMES, M. D.
PHILADELPHIA :
No. 1125 8PRUCK STREET.
L o it r o. :
ALFRED HEATH A CO., Sole Agents for Great Britain,
114 Ebury Street, Eaton Square.
Yearly Subscription, $2.50, invariably in advance. Single numbers, 95 ets.
Entered at the Philadelphia Peet-Otho. aa Seeood-ClaM Mail Matter.
				

